# Breast cancer screening in the Czech Republic: time trends in performance indicators during the first seven years of the organised programme

**DOI:** 10.1186/1471-2458-11-288

**Published:** 2011-05-10

**Authors:** Ondrej Majek, Jan Danes, Miroslava Skovajsova, Helena Bartonkova, Lucie Buresova, Daniel Klimes, Petr Brabec, Pavel Kozeny, Ladislav Dusek

**Affiliations:** 1 Institute of Biostatistics and Analyses, Masaryk University, Kamenice 126/3, 625 00 Brno, Czech Republic; 2 Department of Radiology, First Faculty of Medicine, Charles University in Prague, U Nemocnice 2, 128 08 Prague, Czech Republic; 3 Association of Czech Breast Radiologists, Roskotova 1717/2, 140 44 Prague, Czech Republic; 4 Department of Radiology, Masaryk Memorial Cancer Institute, Zluty kopec 7, 656 53 Brno, Czech Republic; 5 National Reference Centre, Vinohradska 112, 130 00 Prague, Czech Republic

## Abstract

**Background:**

The Czech Breast Cancer Screening Programme (CBCSP) was initiated in September 2002 by establishing a network of accredited centres. The aim of this article is to describe progress in the programme quality over time after the inception of the organised programme.

**Methods:**

The CBCSP is monitored using an information system consisting of three principal components: 1) the national cancer registry, 2) a screening registry collecting data on all screening examinations, further assessments and final diagnoses at accredited programme centres, and 3) administrative databases of healthcare payers. Key performance indicators from the European Guidelines have been adopted for continuous monitoring.

**Results:**

Breast cancer incidence in the Czech Republic has steadily been increasing, however with a growing proportion of less advanced stages. The mortality rate has recently stabilised. The screening registry includes 2,083,285 records on screening episodes between 2002 and 2008. In 2007-2008, 51% of eligible women aged 45-69 were screened. In 2008, the detection rates were 6.1 and 3.7 per 1,000 women in initial and subsequent screening respectively. Corresponding recall rates are 3.9% and 2.2%, however, it is necessary to pay attention to further assessment performed during the screening visits. Benign to malignant open biopsy ratio was 0.1. Of invasive cases detected in screening, 35.6% was less than 10 mm in diameter. Values of early performance indicators, as measured by both crude and standardized estimates, are generally improving and fulfil desirable targets set by European Guidelines.

**Conclusions:**

Mammography screening in the Czech Republic underwent successful transformation from opportunistic prevention to an organised programme. Values of early indicators confirm continuous improvement in different aspects of process quality. Further stimulation of participation through invitation system is necessary to exploit the full potential of screening mammography at the population level.

## Background

Breast cancer is the most frequent malignant neoplasm in women worldwide [1]. In the past, its incidence and mortality in Central and Eastern European countries were significantly lower than in Western Europe. Yet recent changes in reproductive behaviour of women accompanied by significant demographic changes led to a sharp increase in breast cancer incidence in Eastern European countries including the Czech Republic [2]. Stabilisation of mortality from breast cancer can only be achieved through high quality screening associated with adequate treatment of detected tumours [3]. Efficacy of breast cancer screening by mammography in preventing breast cancer deaths was demonstrated in randomised controlled trials. Meta-analysis of Swedish trials showed breast cancer mortality reduction of 29% among women aged 50-69 years [4]. Recent meta-analyses stated mortality reduction to be 15% [5], however, greater protective effect seem to be present in women between 60-69 years [6]. Screening programmes were implemented in many countries worldwide [7,8]. The effect indeed persists in real populations: breast cancer mortality decreased by 16% to 36% in populations invited to screening and women attending at least one screening examination could decrease their risk of death from breast cancer by 24% to 48% [9]. On the other hand, there are also adverse effects associated with breast cancer screening by mammography. Namely these include radiation exposure, pain during mammography, anxiety responses from screening, false-positive and false-negative results, and overdiagnosis [6]. However, there is convincing evidence that, in certain age groups, benefits of mammography screening outweigh its risk. This led to recommendations for screening in United States [10] and Europe [11].

To obtain projected benefits and minimise negative outcomes, the programmes should be implemented with an organised, population-based approach, with quality assurance at all appropriate levels, and in accordance with European Guidelines for Quality Assurance in Breast Cancer Screening and Diagnosis [11,12]. The policy of a screening effort should be documented in a law or an official regulation to qualify as a screening programme [8]. IARC Handbooks of cancer prevention [13] state six characteristics of an organised screening programme: a policy specifying target population, screening method and interval; a defined target population; a team responsible for overseeing screening centres; a decision structure and responsibility for healthcare management; a quality assurance system utilizing relevant data; and monitoring of cancer occurrence in the target population. The highest level of programme organisation, population-based screening, requires that all persons eligible for screening are identified and personally invited to attend a screening examination in each round of screening [8].

The objective of this article is to summarize the implementation and results of the Czech breast cancer screening programme (CBCSP) since 2002. The evaluation of CBCSP is based on a multi-source information system including the monitoring of population cancer burden and early performance indicators of the screening programme. Favourable values of screening performance measures are necessary to have a significant effect on cancer mortality in the future [14]. Therefore, we describe the results of transformation from no-programme opportunistic screening to a non-population based yet highly organised programme by comparison of measured indicators to international guideline targets and published results. To our knowledge, this is one of the first reports from a new EU member state. Compared to previous papers [15-17], this article adds new results on long-term continuous quality improvement after the programme initiation.

## Methods

### The Czech Breast Cancer Screening Programme

In the late 1990s, mammography was performed at more than 130 facilities and screening examinations were claimed as diagnostic. In 2002 an accreditation programme was launched by a directive of the Czech Ministry of Health with accreditation criteria based on the European Guidelines [11]. The programme is administered by the Breast Cancer Screening Committee at the Ministry of Health. The centres are overseen by the Expert Committee on Breast Imaging (*Komise odborníků pro mamární diagnostiku*).

The CBCSP is a nationwide organized programme, currently performed at 67 regularly monitored accredited centres. The target population was defined as women aged 45-69 years. Since 2010 there is no upper age limit, therefore all women from 45 years of age can attend a biennial mammography screening covered by health insurance. Women are referred to screening mammography by their general practitioners (GPs) or gynaecologists on the basis of preventive check-ups, as there is no established centralised system of direct invitation yet. Women outside the target population (over 40 years of age) can undergo screening mammography (upon direct payment of entire cost of the screening examination) and are therefore included in aggregated statistics.

Mammography is performed in two-views (craniocaudal, mediolateral oblique), independent double reading with consensus is recommended. Both screen-film and digital mammography systems are present. Screening centres also act as breast assessment units, necessary further assessment of the findings (magnification, spot mammography, breast ultrasound, etc.) is provided during the screening visit at the centre (one-day diagnostics). Recall to the screening centre is usually employed in case of a double reading discrepancy or for invasive examinations, including core-cut biopsy (majority performed within one week after screening). For reporting of screening and additional imaging results, the BI-RADS system [18] is used and for the evaluation of breast density, typology according to Tabar was included [19]. All of the centres provide core-cut biopsy, special methods (MR mammography and vacuum biopsy) are provided at a narrower network of specialized centres.

**Figure 1:**
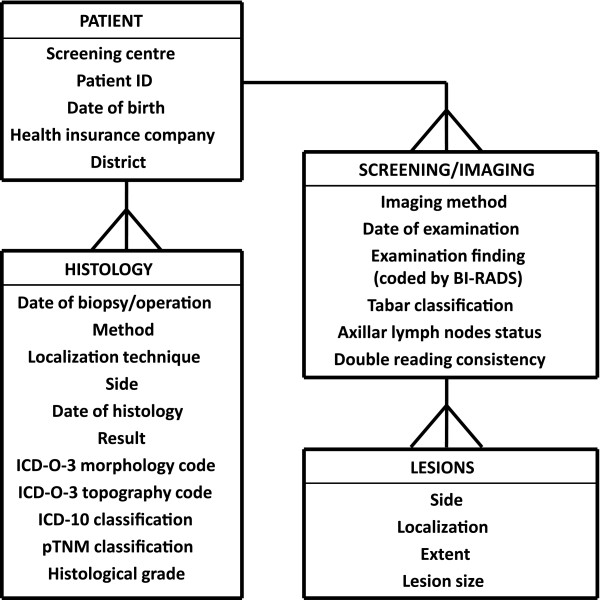
**Structure of a patient record in the Czech Breast Cancer Screening Registry**.

Screening programme data are annually consolidated from local databases of screening centres, and are subsequently stored in a secured central database - the Czech Breast Cancer Screening Registry (Figure 1). Official results containing a description of the screening process and performance monitoring are published annually. Feedback for screening centres is provided using annual reporting of performance indicators.

### Czech National Cancer Registry

The Czech National Cancer Registry (CNCR) was established in 1976. With verified 100% coverage, it contains information on the cancer diagnosis, treatment and survival of all Czech cancer patients. A comprehensive overview of cancer epidemiology in the Czech Republic is available on-line at http://www.svod.cz[20].

### Claims data from healthcare payers

The costs of biennial screening mammography in women from the screening target population are reimbursed by public health insurance in the Czech Republic. Therefore, claims data on mammography are available in data warehouses of healthcare payers provided via centralized exports by the Czech National Reference Centre. These administrative records allow for a description of the utilisation of diagnostic and screening mammography in the Czech female population and to monitor the prevalence of opportunistic screening outside the organised programme.

### Data analysis

Population-based data of CNCR was analysed using standard methods [21] to detect trends in incidence and mortality. The performance of the programme was assessed using the standard set of performance indicators introduced in European Guidelines [11]. The coverage of the target population by screening examination was computed as a ratio between the number of examinations in women aged 45-69 years in previous 24 months and the number of women aged 45-69 in the target population at the end of the period.

Performance of a screening test was assessed using breast cancer detection rates and further assessment rates. Further assessment comprises any additional examination performed until 6 months after screening mammography. Screen-detected cancers comprise all diagnosed breast cancer cases until one year after positive screening. Benign to malignant open biopsy ratio was defined as ratio of the number of women undergoing open biopsy with benign result to the number of women undergoing open biopsy with malignant result. All results were stratified according to the individual screening history of the women: initial or subsequent screening. In order to estimate time-related trends in further assessment rates and detection rates irrespective of changes in age structure and in proportion of initially screened women, standardization [22] was performed. All analyses were performed using Stata/IC 10.1 for Windows [23]. Providers of the utilised data consented with their use for epidemiological research. The study was entirely observational; therefore, no approval from the ethical committee was required.

## Results

### Breast cancer burden in the Czech Republic

The incidence rate of breast cancer has been increasing constantly since the early 1990s (65.5 cases per 100,000 women in 1990 vs. 122.7 in 2007). On the other hand, the mortality rate was stable during the same period (34.2 cases per 100,000 women in 1990 vs. 36.4 in 2007, Figure 2). The peak value of breast cancer incidence can be observed in 2003 after the introduction of an organised screening programme and again in 2007 when the growing programme was reinforced by a pilot project for the invitation of yet-unscreened women (see discussion for description). It is clearly visible particularly in newly introduced women aged 70-74 years (Figure 3). The continuing rise in incidence after 2002 could be attributed to the screening target population, i.e. women aged 45-69 years. The incidence is stable in younger (30-44 years) and older (75+ years) women.

**Figure 2:**
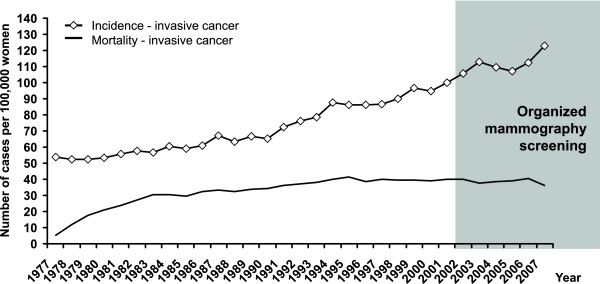
**Time trends of breast cancer incidence and mortality rates in the Czech Republic**. Source of data: Czech National Cancer Registry.

**Figure 3:**
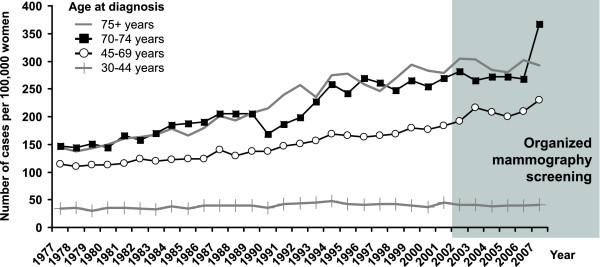
**Time trends in age-specific breast cancer incidence rate**. Source of data: Czech National Cancer Registry.

**Figure 4:**
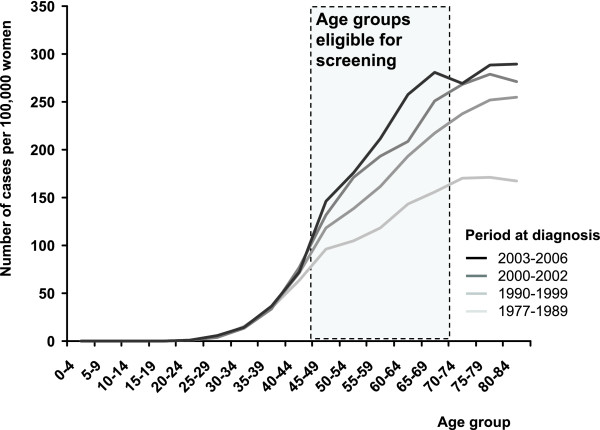
**Comparison of age structure of breast cancer patients groups diagnosed in different time periods**. Source of data: Czech National Cancer Registry.

**Figure 5:**
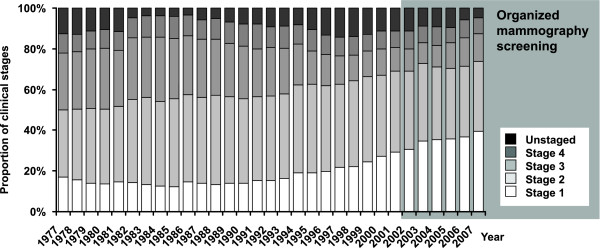
**Time trends in distribution of clinical stages in newly diagnosed breast cancer cases in the Czech Republic**. Source of data: Czech National Cancer Registry.

The rise in incidence rate after 1990 is visible in all postmenopausal age groups. The most recent growth is reduced in the 70-74 age group, due to detection of tumours at a younger age in the screening programme (Figure 4). There is an apparent increase in the proportion of stage I breast cancers which started in the early 1990s and continues after 2002 (Figure 5). This is reflected in increase of early breast cancer rates in the 45-69 age groups. During the same period, we can witness slow decrease in advanced cancer rates (stage III+IV, 50.5 cases per 100,000 women in 1990-1994 vs. 38.1 in 2003-2007, Figure 6).

**Figure 6:**
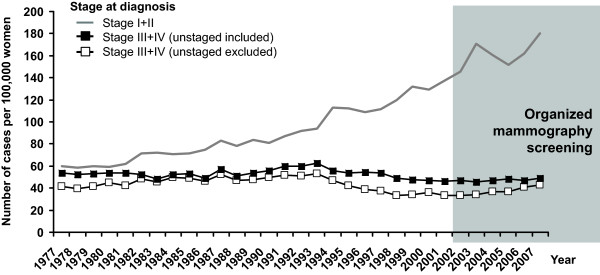
**Time trends of breast cancer incidence rates in women aged 45-69 years, according to clinical stage at diagnosis**. Source of data: Czech National Cancer Registry.

### Results of the organised screening programme

The Czech Breast Cancer Screening Registry includes 2,083,285 records on screening episodes between 2002 and 2008. The 24-month coverage by screening mammography steadily increased over these years (Table 1). The coverage in the age group of 45-69 year-olds reached 51.2% in 2008. In years 2002-2004, women attended programme screening examination for the first time (initial screening). Since then, the proportion of subsequent screenings has been constantly rising and reached 66% in 2008. The age of women attending screenings is also increasing (Table 2). Recruitment is less effective in higher age categories, the coverage decreases from 58.0% at age 45-49 to 42.3% at age 65-69. The screening programme was not open to elderly women (aged over 70) free of charge before 2010. They were only invited as a part of the pilot project in 2007-2008 and the coverage is therefore only 14.5% of this age group (Table 3).

**Table 1:** Performance indicators of the Czech Breast Cancer Screening Programme, according to year of screening, including all age groups (total).

	Year (screening mammography)						
	2002	2003	2004	2005	2006	2007	2008
Number of women screened	10,055	212,537	265,217	317,194	340,564	469,299	468,419
**Estimate of 24 m coverage by examination** **(women aged 45-69)**	**0.6%**	**13.1%**	**27.9%**	**33.8%**	**38.1%**	**44.8%**	**51.2%**
Number of women with detected breast cancer	48	1,053	1,250	1,445	1,570	2,542	2,128
**Breast cancer detection rate**	**4.8**	**5.0**	**4.7**	**4.6**	**4.6**	**5.4**	**4.5**
**Standardized ratio (reference year 2008)**	**0.81**	**0.83**	**0.80**	**0.85**	**0.96**	**0.97**	**1.00**
Number of women undergoing further assessment	1,768	46,976	57,925	56,850	53,580	63,415	60,025
**Further assessment rate**	**17.6%**	**22.1%**	**21.8%**	**17.9%**	**15.7%**	**13.5%**	**12.8%**
**Standardized ratio (reference year 2008)**	**0.90**	**1.20**	**1.21**	**1.14**	**1.10**	**1.01**	**1.00**
Number of women recalled for further assessment	557	12,095	11,725	11,931	10,429	13,977	12,955
**Recall rate**	**5.5%**	**5.7%**	**4.4%**	**3.8%**	**3.1%**	**3.0%**	**2.8%**
Women undergoing open biopsy - benign result	21	504	347	285	194	249	173
Women undergoing open biopsy - malignant result	43	867	1,021	1,259	1,347	2,149	1,794
**Benign to malignant open biopsy ratio**	**0.49**	**0.58**	**0.34**	**0.23**	**0.14**	**0.12**	**0.10**
Number of women with detected breast cancer *(cases preceded by neoadjuvant therapy excluded)*	48	1,031	1,212	1,400	1,522	2,470	2,039
Advanced cases (TNM II+)	17	331	390	440	439	652	525
**Advanced cases proportion**	**35.4%**	**32.1%**	**32.2%**	**31.4%**	**28.8%**	**26.4%**	**25.7%**
Invasive cases	41	933	1,097	1,283	1,370	2,211	1,831
**Invasive cases proportion**	**85.4%**	**90.5%**	**90.5%**	**91.6%**	**90.0%**	**89.5%**	**89.8%**
Node-negative invasive cases	27	572	700	840	905	1,442	1,218
**Proportion among invasive**	**65.9%**	**61.3%**	**63.8%**	**65.5%**	**66.1%**	**65.2%**	**66.5%**
Invasive ≤ 10 mm cases	11	312	360	460	504	770	651
**Proportion among invasive**	**26.8%**	**33.4%**	**32.8%**	**35.9%**	**36.8%**	**34.8%**	**35.6%**

**Table 2:** Time trends in basic characteristics of the population attending the screening programme.

Year screened	NUMBER OF WOMEN SCREENED		Proportion subsequent		AGE SCREENED			
	Initial	Subsequent		5^th^ percentile	25^th^ percentile	Median	75^th^ percentile	95^th^ percentile
**2002**	10,055	0	**0.0%**	45.6	49.2	**53.8**	59.0	66.5
**2003**	212,514	23	**0.0%**	45.7	49.4	**53.9**	59.3	66.7
**2004**	258,635	6,582	**2.5%**	45.4	49.4	**54.2**	59.7	66.9
**2005**	220,897	96,297	**30.4%**	45.5	50.0	**54.9**	60.3	67.1
**2006**	170,717	169,847	**49.9%**	45.5	50.1	**55.1**	60.5	67.2
**2007**	232,509	236,790	**50.5%**	45.3	50.9	**56.9**	63.2	70.6
**2008**	159,273	309,146	**66.0%**	45.3	50.6	**56.1**	61.6	68.0

**Table 3:** Performance indicators of the Czech Breast Cancer Screening Programme, according to age group, women screened in 2008 (total).

	Age group						Total 45-69	Total 50-69
	45-49	50-54	55-59	60-64	65-69	70-74		
Number of women screened	95,883	106,232	105,578	90,394	55,427	4,848	453,514	357,631
**Estimate of 24 m coverage by examination**	**58.0%**	**55.0%**	**51.6%**	**47.5%**	**42.3%**	**14.5%**	**51.2%**	**49.6%**
Number of women with detected breast cancer	254	374	416	577	409	67	2,030	1,776
**Breast cancer detection rate**	**2.6**	**3.5**	**3.9**	**6.4**	**7.4**	**13.8**	**4.5**	**5.0**
Number of women undergoing further assessment	20,881	14,923	10,318	7,174	3,959	409	57,255	36,374
**Further assessment rate**	**21.8%**	**14.0%**	**9.8%**	**7.9%**	**7.1%**	**8.4%**	**12.6%**	**10.2%**
Number of women recalled for further assessment	3,652	2,913	2,421	2,149	1,291	162	12,426	8,774
**Recall rate**	**3.8%**	**2.7%**	**2.3%**	**2.4%**	**2.3%**	**3.3%**	**2.7%**	**2.5%**
Women undergoing open biopsy - benign result	50	40	27	31	21	1	169	119
Women undergoing open biopsy - malignant result	211	315	368	481	334	58	1,709	1,498
**Benign to malignant open biopsy ratio**	**0.24**	**0.13**	**0.07**	**0.06**	**0.06**	**0.02**	**0.10**	**0.08**
Number of women with detected breast cancer *(cases preceded by neoadjuvant therapy excluded)*	239	352	401	555	399	65	1,946	1,707
Advanced cases (TNM II+)	63	98	104	131	95	27	491	428
**Advanced cases proportion**	**26.4%**	**27.8%**	**25.9%**	**23.6%**	**23.8%**	**41.5%**	**25.2%**	**25.1%**
Invasive cases	216	315	343	508	360	63	1,742	1,526
**Invasive cases proportion**	**90.4%**	**89.5%**	**85.5%**	**91.5%**	**90.2%**	**96.9%**	**89.5%**	**89.4%**
Node-negative invasive cases	131	196	238	342	255	38	1,162	1,031
**Proportion among invasive**	**60.6%**	**62.2%**	**69.4%**	**67.3%**	**70.8%**	**60.3%**	**66.7%**	**67.6%**
Invasive ≤ 10 mm cases	70	110	119	179	146	19	624	554
**Proportion among invasive**	**32.4%**	**34.9%**	**34.7%**	**35.2%**	**40.6%**	**30.2%**	**35.8%**	**36.3%**

**Figure 7:**
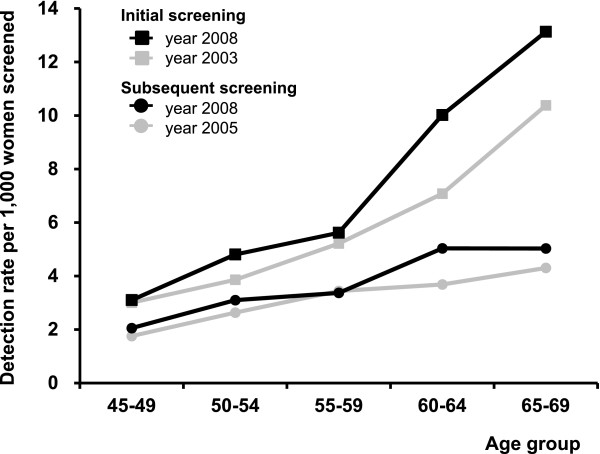
**Breast cancer detection rate: age-specific comparison of time periods**.

The crude breast cancer detection rate has been changing a little during first years of the programme (Table 1). Over years and age subgroups, the detection rate was always higher in initially screened women (Tables 4, 5) than in subsequently screened women (Tables 6, 7). The detection rate increases with age in initial (3.1-15.1 detected cases per 1,000 women screened, Table 5) and subsequent (2.1-11.7 detected cases per 1,000 women screened, Table 7) screenings. Bearing the rapidly changing population structure in mind, it is therefore more informative to study development of age-specific rates in initial and subsequent screenings (Figure 7) or summarize them using a standardized rate ratio (Table 1). Both show evidence for constantly improving detection rates after the organisation of the screening programme.

**Table 4:** Performance indicators of the Czech Breast Cancer Screening Programme, according to year of screening, including all age groups (initial screening).

	Year (screening mammography)						
	2002	2003	2004	2005	2006	2007	2008
Number of women screened	10,055	212,514	258,635	220,897	170,717	232,509	159,273
Number of women with detected breast cancer	48	1,053	1,220	1,153	935	1,662	974
**Breast cancer detection rate**	**4.8**	**5.0**	**4.7**	**5.2**	**5.5**	**7.1**	**6.1**
Number of women undergoing further assessment	1,768	46,973	56,961	44,919	33,135	39,921	30,239
**Further assessment rate**	**17.6%**	**22.1%**	**22.0%**	**20.3%**	**19.4%**	**17.2%**	**19.0%**
Number of women recalled for further assessment	557	12,095	11,535	9,735	6,496	8,888	6,246
**Recall rate**	**5.5%**	**5.7%**	**4.5%**	**4.4%**	**3.8%**	**3.8%**	**3.9%**
Women undergoing open biopsy - benign result	21	504	343	226	135	166	90
Women undergoing open biopsy - malignant result	43	867	992	994	787	1,382	784
**Benign to malignant open biopsy ratio**	**0.49**	**0.58**	**0.35**	**0.23**	**0.17**	**0.12**	**0.11**
Number of women with detected breast cancer *(cases preceded by neoadjuvant therapy excluded)*	48	1,031	1,182	1,116	905	1,610	924
Advanced cases (TNM II+)	17	331	384	367	284	465	277
**Advanced cases proportion**	**35.4%**	**32.1%**	**32.5%**	**32.9%**	**31.4%**	**28.9%**	**30.0%**
Invasive cases	41	933	1,072	1,023	823	1,459	850
**Invasive cases proportion**	**85.4%**	**90.5%**	**90.7%**	**91.7%**	**90.9%**	**90.6%**	**92.0%**
Node-negative invasive cases	27	572	682	644	525	909	535
**Proportion among invasive**	**65.9%**	**61.3%**	**63.6%**	**63.0%**	**63.8%**	**62.3%**	**62.9%**
Invasive ≤ 10 mm cases	11	312	350	348	288	441	275
**Proportion among invasive**	**26.8%**	**33.4%**	**32.6%**	**34.0%**	**35.0%**	**30.2%**	**32.4%**

**Table 5:** Performance indicators of the Czech Breast Cancer Screening Programme, according to age group, women screened in 2008 (initial screening).

	Age group						Total 45-69	Total 50-69
	45-49	50-54	55-59	60-64	65-69	70-74		
Number of women screened	54,495	26,214	26,694	24,452	16,067	3,047	147,922	93,427
Number of women with detected breast cancer	169	126	150	245	211	46	901	732
**Breast cancer detection rate**	**3.1**	**4.8**	**5.6**	**10.0**	**13.1**	**15.1**	**6.1**	**7.8**
Number of women undergoing further assessment	14,188	5,097	3,749	2,998	1,808	306	27,840	13,652
**Further assessment rate**	**26.0%**	**19.4%**	**14.0%**	**12.3%**	**11.3%**	**10.0%**	**18.8%**	**14.6%**
Number of women recalled for further assessment	2,430	1,007	845	901	626	124	5,809	3,379
**Recall rate**	**4.5%**	**3.8%**	**3.2%**	**3.7%**	**3.9%**	**4.1%**	**3.9%**	**3.6%**
Women undergoing open biopsy - benign result	35	18	8	14	11	1	86	51
Women undergoing open biopsy - malignant result	135	101	132	194	161	38	723	588
**Benign to malignant open biopsy ratio**	**0.26**	**0.18**	**0.06**	**0.07**	**0.07**	**0.03**	**0.12**	**0.09**
Number of women with detected breast cancer *(cases preceded by neoadjuvant therapy excluded)*	159	115	144	233	205	44	856	697
Advanced cases (TNM II+)	47	34	45	72	55	19	253	206
**Advanced cases proportion**	**29.6%**	**29.6%**	**31.3%**	**30.9%**	**26.8%**	**43.2%**	**29.6%**	**29.6%**
Invasive cases	146	106	129	216	189	42	786	640
**Invasive cases proportion**	**91.8%**	**92.2%**	**89.6%**	**92.7%**	**92.2%**	**95.5%**	**91.8%**	**91.8%**
Node-negative invasive cases	83	68	85	136	124	24	496	413
**Proportion among invasive**	**56.8%**	**64.2%**	**65.9%**	**63.0%**	**65.6%**	**57.1%**	**63.1%**	**64.5%**
Invasive ≤ 10 mm cases	47	32	42	64	73	10	258	211
**Proportion among invasive**	**32.2%**	**30.2%**	**32.6%**	**29.6%**	**38.6%**	**23.8%**	**32.8%**	**33.0%**

**Table 6:** Performance indicators of the Czech Breast Cancer Screening Programme, according to year of screening, including all age groups (subsequent screening).

	Year (screening mammography)				
	2004	2005	2006	2007	2008
Number of women screened	6,582	96,297	169,847	236,790	309,146
Number of women with detected breast cancer	30	292	635	880	1,154
**Breast cancer detection rate**	**4.6**	**3.0**	**3.7**	**3.7**	**3.7**
Number of women undergoing further assessment	964	11,931	20,445	23,494	29,786
**Further assessment rate**	**14.6%**	**12.4%**	**12.0%**	**9.9%**	**9.6%**
Number of women recalled for further assessment	190	2,196	3,933	5,089	6,709
**Recall rate**	**2.9%**	**2.3%**	**2.3%**	**2.1%**	**2.2%**
Women undergoing open biopsy - benign result	4	59	59	83	83
Women undergoing open biopsy - malignant result	29	265	560	767	1,010
**Benign to malignant open biopsy ratio**	**0.14**	**0.22**	**0.11**	**0.11**	**0.08**
Number of women with detected breast cancer *(cases preceded by neoadjuvant therapy excluded)*	30	284	617	860	1,115
Advanced cases (TNM II+)	6	73	155	187	248
**Advanced cases proportion**	**20.0%**	**25.7%**	**25.1%**	**21.7%**	**22.2%**
Invasive cases	25	260	547	752	981
**Invasive cases proportion**	**83.3%**	**91.5%**	**88.7%**	**87.4%**	**88.0%**
Node-negative invasive cases	18	196	380	533	683
**Proportion among invasive**	**72.0%**	**75.4%**	**69.5%**	**70.9%**	**69.6%**
Invasive ≤ 10 mm cases	10	112	216	329	376
**Proportion among invasive**	**40.0%**	**43.1%**	**39.5%**	**43.8%**	**38.3%**

**Table 7:** Performance indicators of the Czech Breast Cancer Screening Programme, according to age group, women screened in 2008 (subsequent screening).

	Age group						Total 45-69	Total 50-69
	45-49	50-54	55-59	60-64	65-69	70-74		
Number of women screened	41,388	80,018	78,884	65,942	39,360	1,801	305,592	264,204
Number of women with detected breast cancer	85	248	266	332	198	21	1,129	1,044
**Breast cancer detection rate**	**2.1**	**3.1**	**3.4**	**5.0**	**5.0**	**11.7**	**3.7**	**4.0**
Number of women undergoing further assessment	6,693	9,826	6,569	4,176	2,151	103	29,415	22,722
**Further assessment rate**	**16.2%**	**12.3%**	**8.3%**	**6.3%**	**5.5%**	**5.7%**	**9.6%**	**8.6%**
Number of women recalled for further assessment	1,222	1,906	1,576	1,248	665	38	6,617	5,395
**Recall rate**	**3.0%**	**2.4%**	**2.0%**	**1.9%**	**1.7%**	**2.1%**	**2.2%**	**2.0%**
Women undergoing open biopsy - benign result	15	22	19	17	10	0	83	68
Women undergoing open biopsy - malignant result	76	214	236	287	173	20	986	910
**Benign to malignant open biopsy ratio**	**0.20**	**0.10**	**0.08**	**0.06**	**0.06**	**0.00**	**0.08**	**0.07**
Number of women with detected breast cancer *(cases preceded by neoadjuvant therapy excluded)*	80	237	257	322	194	21	1,090	1,010
Advanced cases (TNM II+)	16	64	59	59	40	8	238	222
**Advanced cases proportion**	**20.0%**	**27.0%**	**23.0%**	**18.3%**	**20.6%**	**38.1%**	**21.8%**	**22.0%**
Invasive cases	70	209	214	292	171	21	956	886
**Invasive cases proportion**	**87.5%**	**88.2%**	**83.3%**	**90.7%**	**88.1%**	**100.0%**	**87.7%**	**87.7%**
Node-negative invasive cases	48	128	153	206	131	14	666	618
**Proportion among invasive**	**68.6%**	**61.2%**	**71.5%**	**70.5%**	**76.6%**	**66.7%**	**69.7%**	**69.8%**
Invasive ≤ 10 mm cases	23	78	77	115	73	9	366	343
**Proportion among invasive**	**32.9%**	**37.3%**	**36.0%**	**39.4%**	**42.7%**	**42.9%**	**38.3%**	**38.7%**

**Figure 8:**
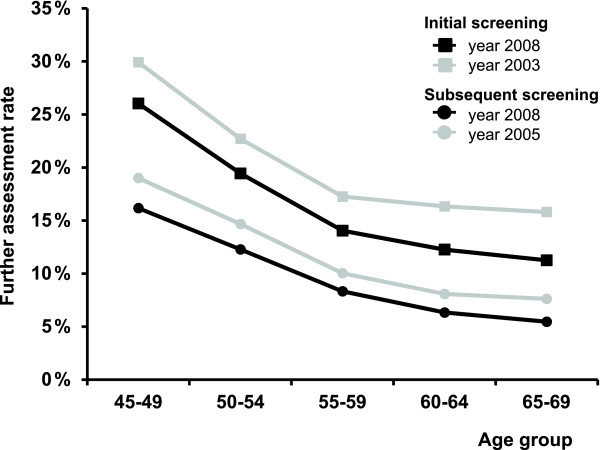
**Further assessment rate: age-specific comparison of time periods**.

The further assessment rate has been decreasing since 2003 to 12.8% in 2008 (Table 1). This indicator is also subject to trends in age structure and the proportion of subsequent examinations. This rate decreases with age in initial (26.0%-10.0%, Table 5) and subsequent (16.2%-5.7%, Table 7) screenings, being larger in initial screening. Improvement (i.e. decrease) in further assessment rates is clear from both age-specific (Figure 8) and standardized indicators (Table 1). Trends in the recall rate are very similar to those in the further assessment rate. Only about 20-30% of women with positive results of screening are being recalled back to the screening centre. The rest of the women undergo further assessment on the day of the screening visit. The benign to malignant open biopsy ratio is also constantly improving (0.49 in 2002 vs. 0.10 in 2008). The ratio is similar in initially and subsequently screened women. The youngest age groups experience a slightly increased ratio (Tables 5, 7).

About 30% of detected breast cancer cases are advanced (TNM stage II+, i.e. involvement of lymph nodes and/or distant metastases) in initial screenings, and this proportion decreases to about 20% in subsequent screenings (Tables 4, 6). About 90% of detected cases are invasive in both initial and subsequent screenings. Of these, 60-70% of the findings are without involvement of lymph nodes (this proportion is higher in subsequent screenings). The primary tumour in about 30-40% of invasive cases is less than 10 mm in diameter (this proportion is higher in subsequent screenings).

## Discussion

A recent study [9] reviewed current evidence on the effectiveness of mammography screening in real populations. The authors identified reports from eight European countries, where mammography screening lead to an eventual decrease in breast cancer mortality. National programmes included Finland [24], Iceland [25], the Netherlands [26] and the United Kingdom [27]. Regionally organised programmes included Denmark [28], Italy [29], Spain [30] and Sweden [31,32]. All of these countries implemented organised screening programmes in the 1980s or early 1990s. Organised screening policies in these countries include the specification of covered age groups, screening interval and detection methods [7]. Programmes include quality assurance systems [33] that utilise data on screening process and also have access to cancer registry data [34].

The CBCSP has been implemented to comprise such organisational aspects, as recommended by the IARC working group. We made comprehensive use of three valuable data sources (cancer registry, cancer screening registry, and the data warehouse of healthcare payers) to continuously monitor the success of the programme. The following discussion compares observed values of early performance indicators with published results and European Guidelines targets [11], which are based on the results of randomized trials and successful screening programmes.

### Epidemiology

In addition to the performance monitoring, the Czech screening programme is supported with highly representative epidemiology cancer registry covering the whole population since 1977. Breast cancer incidence has been rising steadily since 1980s. However, further increase in mortality has been arrested after 1995, similarly to other member states of the European Union [3]. The stabilized breast cancer mortality in the Czech Republic can be at least partially attributed to the improving stage distribution of newly diagnosed breast cancer cases and mild decrease in rate of advanced tumours. Nevertheless, part of the increase in early tumours rate is attributable to overdiagnosis - screen-detected cancers that would not have surfaced clinically during the woman's lifetime [35]. Estimate of the proportion of overdiagnosed tumours ranges to over 50% [36]; however, most of the estimates vary from 1% to 10% [6]. Very gradual expansion of opportunistic and organised screening does not yet allow us to estimate extent of overdiagnosis precisely using epidemiology data; therefore, we have concentrated on assessment of early performance indicators. As their values of detection rates and DCIS proportion comply with European Guidelines targets quite well (see bellow), we do not assume overdiagnosis to markedly excess values seen in clinical and epidemiological studies abroad.

### Coverage

The current Czech system of recruitment through GPs or gynaecologists, reinforced by media campaigns and recall for subsequent screenings by screening centres was able to achieve a modest coverage of 50%. The advantage of this setup is the primary care physician's full knowledge of the patient's medical history and preferences, which enables the proper tailoring of an individual preventive strategy. Nevertheless, participation rates in successful population-based programmes approach the European Guidelines target of 70% (e.g. Spain [37], United Kingdom [38], Denmark [39]), or even exceed them (Finland [40]). However, the invitation may also fail to achieve the stated target (e.g. decentralized invitation in Hungary [16], invitation without appointment in Luxembourg [41]) - it is therefore necessary to properly plan, implement and monitor the invitation process.

### Pilot project

That is why in 2007-2008 a pilot project of centralised invitation of non-attending women was undertaken. The project was carried out by the General Health Insurance Company (GHIC). GHIC is the principal provider of health insurance in the Czech Republic, with more than 6.5 million clients (about two thirds of the Czech population). A total number of 598,637 women aged between 45 and 74 years were invited for screening mammography (the invited women had not undergone mammography examination during the last three years). The women invited to the pilot project were screened between July 2007 and February 2008. Overall, 107,264 women (i.e., 18% of those invited) were screened at 60 mammography screening facilities. As regards the target population (45-69 years), 491,294 women were invited and 16.4% of them were screened. Participation rate was higher in older women (24.7%).

Despite the relatively low participation rate in the project itself, the project helped to substantially increase coverage in the target population (from 38.1% in 2006 to 51.2% in 2008), especially in elder women. Furthermore, the pilot project also invited women aged 70-74 and the outcomes of the screening in this group are visible in the epidemiology data. The pilot project confirmed a high sensitivity and specificity of mammography in these elderly groups and led to the extension of the age groups targeted by the Czech screening programme in 2010.

### Opportunistic activities

Screening activities occurring outside the programme or before its inception are referred to as 'opportunistic' screening. Opportunistic screening may fail to exploit the full potential of mammography to prevent deaths from breast cancer [42]. A possible explanation includes less sensitivity [43] and subsequently less-favourable prognostic features of detected tumours [44]. It is less cost-effective, mainly due to higher cost of diagnostic mammography and overuse of additional imaging in an unorganized setting [45,46]. Dissemination of organised mammography screening can increase programme coverage [17] and also attract more disadvantaged women [47]. It is therefore advisable to promote the highest level of organisation possible, with close monitoring of performance indicators [14].

Intensified nationwide organisation led to an increase in opportunistic mammography activities in Hungary [17]. Yet Jensen [48] concluded that, in Denmark, women not attending an organised programme did not seek the service elsewhere and proportion of women screened outside the programme was 1-4% in Danish counties. According to monitoring performed by the Czech healthcare payers, the non-programme screening was quite prevalent in the Czech Republic before the onset of the organized programme. In 2002, the proportion of women undergoing non-programme examination was over 15% and approached 25% in some regions. In the following years, the overall proportion has been decreasing below 10%; however, it still remains high in some regions.

### Sensitivity of the programme

Important early indirect measures of test and programme sensitivity include detection rate, stage distribution of detected cancers and incidence of interval cancers [11,14].

The presence of opportunistic screening and gradual recruitment into organised screening make it difficult to estimate incidence rates expected in the target population in the absence of screening (background incidence), which is necessary to interpret detection and interval cancer rates. Anyway, a rough estimate could be acquired by averaging pre-programme breast cancer rates. The resulting ratio of detection rate to background incidence rate in initial screening for women aged 50-69 years is 4.1, which is in accordance with European Guidelines and similar to values observed in European pilot projects [49].

Easily observable measures of stage distribution are defined in European Guidelines. The proportion of advanced tumours and the proportion of small invasive tumours achieved the desirable levels given by European Guidelines. On the other hand, the proportion of node-negative invasive tumours keeps staying below acceptable level. However, modern pathology techniques may lead to increased detection of node metastases of lesser clinical significance [50,51] and targets might need to be restated in light of the new epidemiological data.

Precise estimation of interval cancer rates has not yet been possible in the Czech screening, due to the non-existing direct individual link between the cancer registry and the cancer screening registry. Prediction of the rate is possible, e.g. using the Markov model [52], however, we don't consider this approach for continuous monitoring of performance indicators.

### Safety and efficiency of the programme

A specific feature of the CBCSP is one-day diagnostics. Providing final results of a screening examination to the women during the screening visit definitely prevents a great amount of adverse psychological consequences drawn by the screening programme [53] and provides a sufficient level comfort. Yet, it might be quite demanding for the staff of the screening centres and might increase the further assessment rate, because the women are readily available at the screening centre during the mammogram assessment. Indeed, despite the continuous decrease in time, further assessment remains 2-3 times higher than in Western and Northern European countries [54]. Recall rates are nevertheless similar, as only some of women with further assessment are actually recalled back to the screening centre.

The high positive predictive value of further assessment is provided by the successful adoption of preoperative diagnostics with core biopsy. This part of the screening process is reflected by a benign to malignant open biopsy ratio, one of the key indicators in the European Guidelines. Results in the CBCSP achieved the desirable target in the Guidelines and became fully comparable to long operating population-based screening programmes, e.g. in Finland and Italy [40,55].

## Conclusions

The transformation from opportunistic prevention to an organised programme facilitated continuous improvement in the quality of offered mammography screening examinations. Most performance indicators reach targets set by European Guidelines and observed in successful population-based programmes around Europe. This promises effectiveness, safety and efficiency similar to randomized clinical trials, which justifies the enormous investment into programme initiation and operation. The important task now is to implement addressed invitations to the screenings and to institute a system for monitoring the impact of cancer screening on population epidemiology, including examination of the possible risk of overdiagnosis.

## Competing interests

The authors declare that they have no competing interests.

## Authors' contributions

OM, LB and LD designed and performed the statistical analysis. JD, MS and HB participated in the design of the study and were responsible for interpretation of the programme results from the radiological viewpoint. DK and PB were responsible for design and administration of the Czech Breast Cancer Screening Registry. PK coordinated providing of healthcare payers data. OM was responsible for coordination of writing of the manuscript. All authors read and approved the final manuscript.

## Pre-publication history

The pre-publication history for this paper can be accessed here:

http://www.biomedcentral.com/1471-2458/11/288/prepub
